# Overexpression of F-Box Nictaba Promotes Defense and Anthocyanin Accumulation in *Arabidopsis thaliana* After *Pseudomonas syringae* Infection

**DOI:** 10.3389/fpls.2021.692606

**Published:** 2021-07-29

**Authors:** Andrea Romero-Pérez, Maarten Ameye, Kris Audenaert, Els J. M. Van Damme

**Affiliations:** ^1^Laboratory of Biochemistry and Glycobiology, Department of Biotechnology, Ghent University, Ghent, Belgium; ^2^Laboratory of Applied Mycology and Phenomics, Department of Plants and Crops, Ghent University, Ghent, Belgium

**Keywords:** glycosylation, lectin, F-Box Nictaba, *Pseudomonas*, plant-pathogen interaction

## Abstract

*Pseudomonas syringae* pv. *tomato* DC3000 (*Pst* DC3000) is a well-known pathogen and model organism used to study plant-pathogen interactions and subsequent plant immune responses. Numerous studies have demonstrated the effect of *Pst* DC3000 on *Arabidopsis* plants and how type III effectors are required to promote bacterial virulence and pathogenesis. F-Box Nictaba (encoded by *At2g02360*) is a stress-inducible lectin that is upregulated in *Arabidopsis thaliana* leaves after *Pst* DC3000 infection. In this study, a flood inoculation assay was optimized to check the performance of transgenic *Arabidopsis* seedlings with different expression levels of F-Box Nictaba after bacterial infection. Using a combination of multispectral and fluorescent imaging combined with molecular techniques, disease symptoms, transcript levels for F-Box Nictaba, and disease-related genes were studied in *Arabidopsis* leaves infected with two virulent strains: *Pst* DC3000 and its mutant strain, deficient in flagellin Δ*fliC*. Analyses of plants infected with fluorescently labeled *Pst* DC3000 allowed us to study the differences in bacterial colonization between plant lines. Overexpression plants showed a reduced bacterial content during the later stages of the infection. Our results show that overexpression of F-Box Nictaba resulted in reduced leaf damage after bacterial infections, whereas knockdown and knockout lines were not more susceptible to *Pseudomonas* infection than wild-type plants. In contrast to wild-type and knockout plants, overexpressing lines for F-Box Nictaba revealed a significant increase in anthocyanin content, better efficiency of photosystem II (Fv/Fm), and higher chlorophyll content after *Pst* DC3000 infection. Overexpression of F-Box Nictaba coincided with increased expression of salicylic acid (SA) related defense genes, confirming earlier data that showed that F-Box Nictaba is part of the SA-dependent defense against *Pst* DC3000 infection. Knockout lines yielded no discernible effects on plant symptoms after *Pseudomonas* infection suggesting possible gene redundancy between F-Box Nictaba genes.

## Introduction

Lectins are defined as proteins that selectively recognize and reversibly bind to specific carbohydrate structures without making structural changes to the carbohydrate moiety. These carbohydrate-binding proteins are present in all kingdoms of life and have been studied extensively in animals and plants, but also occur in bacteria, fungi, and viruses (Peumans and Van Damme, 1995; Lannoo and Van Damme, 2010; Van Holle and Van Damme, 2018). In plants, two major groups of lectins are distinguished based on their subcellular localization and expression pattern. The classical lectins are constitutively expressed and mainly reside in the vacuole, in contrast to the stress-inducible lectins located in the nucleus and the cytoplasm. The latter group of lectins is expressed at a basal level under normal growth conditions, but their expression is upregulated when plants face unfavorable conditions, such as biotic or abiotic stresses (Van Holle and Van Damme, 2018). Consequently, these lectins have been suggested to be involved in cell signaling events (Van Damme et al., 2004). Understanding the protein-carbohydrate interactions in plant cells is crucial to discern the mode of action of lectins (Lannoo and Van Damme, 2010).

Proteins are considered lectins if they contain one or more lectin domains. Chimeric lectins are composed of one or more lectin domains fused to another protein domain. Depending on the sequence of the lectin domains, the whole group of plant lectins is divided into 12 different families (Tsaneva and Van Damme, 2020). One of these lectin families is named after the *Nicotiana tabacum* agglutinin, better known as Nictaba, a dimeric lectin composed of 19 kDa subunits (Chen et al., 2002). This lectin was one of the first proteins to be classified as a stress-inducible lectin. Its presence in tobacco leaves was not detectable when plants are grown under normal conditions, but Nictaba levels were upregulated after jasmonate treatment, caterpillar attack, and cold treatment (Chen et al., 2002; Vandenborre et al., 2009; Lannoo and Van Damme, 2010; Delporte et al., 2011). Nictaba-like proteins are widespread in the plant kingdom and reported in the transcriptome of *Arabidopsis thaliana, tomato*, cucumber, and rice, among other species (Van Holle et al., 2017). Different studies provide evidence for the role of Nictaba-like proteins in plant defense. Overexpression of the *Arabidopsis* Nictaba homolog PP2-A1 (*At4g19840*) improved plant tolerance against insect attack. PP2-A1 also presents anti-fungal properties, and its expression is upregulated after ethylene treatment and *Pseudomonas* infection (Beneteau et al., 2010; Zhang et al., 2011; Lee et al., 2014). The overexpression of the Nictaba homolog AN4 (*At1g31200*) in *Arabidopsis thaliana* confers higher tolerance against *Pseudomonas syringae* infection (Eggermont et al., 2018). In *Arabidopsis*, the Nictaba domain can be combined with different protein domains, such as the AIG1 domain, the TIR domain, or the F-Box domain (Delporte et al., 2015). The *Arabidopsis thaliana* genome presents 19 genes that encode chimeric proteins composed of F-Box and Nictaba domains (Eggermont et al., 2017). These F-Box Nictaba related sequences show between 22 and 90% sequence identity at the amino acid level.

F-box proteins contain an F-box motif consisting of ~50 amino acids that act as a site for protein–protein interaction for ubiquitin-mediated proteolysis (Kirpreos and Pagano, 2000). The F-box (FBX) genes represent one of the largest and more diverse superfamilies of genes in the plant kingdom (Hua et al., 2011). They are present in different eukaryotes, such as humans, *Drosophila*, yeast, *Caenorhabditis elegans*, or *Arabidopsis thaliana* (Gagne et al., 2002). The *Arabidopsis thaliana* genome contains almost 700 predicted FBX genes, representing around 2.7% of the *Arabidopsis* genome (Gagne et al., 2002). Multiple studies have proven that F-Box proteins are important for different biological and physiological processes in plants, such as self-incompatibility, control of the circadian clock, photomorphogenesis, and flowering time (Eckardt, 2004; Stefanowicz et al., 2015). Furthermore, these proteins are involved in the plant response to biotic or abiotic stresses (Maldonado-Calderón et al., 2012; Stefanowicz et al., 2016).

F-Box Nictaba (*At2g02360*) represents a 38 kDa nucleocytoplasmic protein and has been studied especially for its role in the plant response against different abiotic and biotic stresses. Glycan array analyses revealed that F-Box Nictaba exhibits specificity toward N- and O-glycans containing (poly) N-acetyllactosamine (Galβ1-3GlcNAc and Galβ1-4GlcNAc) structures and poly-LacNAc structures as well as Lewis A [Galβ1-3(Fucα1-4)GlcNAc], Lewis X [Galβ14(Fucα1-3)GlcNAc], Lewis Y [Fucα1-2Galβ1-4(Fucα13)GlcNAc] and type-1 B motifs [Galα1-3(Fucα1-2)Galβ13GlcNAc] (Stefanowicz et al., 2012). Experiments with 5-week-old wild-type *Arabidopsis thaliana* plants showed an up-regulation of the transcript levels for F-Box Nictaba after *Pseudomonas syringae* pv. *tomato* DC3000 infection and after treatment with the plant hormone salicylic acid. Five-week-old plants overexpressing F-Box Nictaba yielded reduced infection symptoms after bacterial infection, suggesting a role for F-Box Nictaba in defense of *Arabidopsis thaliana* plants against *Pseudomonas syringae* (Stefanowicz et al., 2016).

This study aimed to provide more insights into the role of F-Box Nictaba in the plant response to virulent *Pseudomonas* strains. Wild-type Col-0 *Arabidopsis* plants and transgenic lines with different levels of F-Box Nictaba expression (overexpression, knockdown, and CRISPR-generated knockout lines) were infected with different *Pseudomonas syringae* strains. Disease symptoms and transcript levels for F-Box Nictaba and disease-related genes were analyzed in *Arabidopsis* leaves after infection with the virulent strains *Pseudomonas syringae* pv. *tomato* DC3000 strain (*Pst* DC3000) or a mutant strain of *Pst* DC3000 deficient in flagellin Δ*fliC*. In addition to leaf damage, other plant parameters such as the efficiency of photosystem II and chlorophyll content were investigated. The F-Box Nictaba knockout lines were equally susceptible to infection than wild-type plants. However, F-Box Nictaba overexpressing lines were more tolerant of *Pseudomonas* infection since seedlings overexpressing F-Box Nictaba clearly showed reduced leaf damage than wild-type seedlings. Multispectral and fluorescence imaging of *Pst* DC3000 infected plants showed that transgenic lines overexpressing F-Box Nictaba presented an increased anthocyanin content compared with mock-treated plants. Our results indicate that F-Box Nictaba contributes to the defense response of *Arabidopsis thaliana* against *Pseudomonas* infection by reducing the infection symptoms. The symptom reduction is related to increased levels of anthocyanins in plants overexpressing F-Box Nictaba. Anthocyanins are compounds related to antioxidant properties and help the plant cope with the oxidative stress provoked by bacterial infection. The overexpression of F-Box Nictaba could help the plant to deal with the oxidative stress induced during plant defense in response to *Pseudomonas* infection.

## Materials and Methods

### Plant Material and Growth Conditions

Seeds of wild-type (WT) *Arabidopsis thaliana* ecotype Columbia 0 were kindly provided by Professor Richard Strasser (Department of Applied Genetics and Cell Biology, University of Natural Resources and Life Sciences, Vienna, Austria). Seeds of homozygous lines overexpressing F-Box Nictaba (OE6 and OE4) were available in the laboratory (Stefanowicz et al., 2016). The lines OE6 and OE4 were selected from a pool of overexpression lines based on the level of overexpression of F-Box Nictaba. Seeds of the SALK T-DNA insertion mutant line associated with the locus *At2g02360* (SALK_085735C), further referred to as the knockdown line (KD) and the mutant *npr1-1*, were purchased from the European Arabidopsis Stock Center (NASC, University of Nottingham, UK). The F-Box Nictaba gene was upregulated after SA treatment, whereas NPR1 acts as a regulator of SA-dependent signaling (Chen et al., 2021). Since this study also investigates the relation between F-Box Nictaba and SA-dependent defense responses, *npr1-1* was included in the experiments. F-Box Nictaba knockout lines were obtained using the CRISPR-Cas technique in this study.

To grow *in vitro* plantlets, seeds were sterilized by washing in 70% (v/v) ethanol for 2 min, washing in 5% (v/v) sodium hypochlorite (Sigma-Aldrich, St. Louis, MO) for 10 min, followed by rinsing up to eight times with sterile bidestilled water. After sterilization, 30 seeds were placed on Murashige and Skoog (MS) medium [4.3 g/L Murashige and Skoog supplemented with modified vitamins (Duchefa, Haarlem, The Netherlands), 30 g/L sucrose (Duchefa), adjusted to pH 5.7 and 8 g/L plant agar (Duchefa)]. Subsequently, plates (100 mm Ø, Greiner Bio One International GmbH, Oberösterreich, Austria) were placed at 4°C in the dark for 3 days to break the dormancy and allow the stratification of the seeds. Afterward, plates were transferred to a growth chamber set at 21°C with a 16/8 h light/dark photoperiod. In order to grow *in vivo* plants, 2-week-old *Arabidopsis thaliana* plants grown on MS plates were transferred to artificial soil (AS Jiffy Products, Drobak, Norway, 44 mm Ø) (AS Jiffy Products). After transferring to soil substrate, plants were protected with plastic foil for a week to let them adapt to the new growth conditions.

### F-Box Nictaba Knockout Lines by CRISPR-Cas Technology

The guide sequence of sgRNA was designed using CRISPR-P 2.0 and consists of 20 nucleotides, followed by the PAM sequence with six nucleotides. CRISPR vectors (pEN-Sa-Chimera and pDe-Sa-CAS9) were supplied by Prof. Dr. Holger Puchta (Botanical Institute II, Karlsruhe Institute of Technology, Karlsruhe, Germany). Gateway compatible cloning of single sgRNAs was performed according to the adapted protocol provided by Professor Dr. Holger Puchta. The single sgRNA was introduced in the entry vector (pEn-Sa-Chimera) using the BbsI restriction enzyme for digestion of the vector and T4 ligase for the ligation of the sgRNA sequence. The Gateway technology enabled to clone of the pEn-Sa-Chimera vector into the destination vector (pEn-Sa-CAS9) with the help of the LR Clonase II enzyme. The destination vector was introduced in *Agrobacterium tumefaciens* C58C1 pGV4000 using cold-shock transformation of competent *Agrobacterium* cells. The transformation of the *Agrobacterium* colonies was checked using colony PCR.

Eight-week-old wild-type *Arabidopsis* plants were transformed using the floral dip method (Clough and Bent, 1998). T1 seeds were sown on MS plates containing kanamycin (75 μg/mL) as a selective agent. Seedlings were transferred to artificial potting soil (Jiffy, 44mm Ø) (AS Jiffy Products, Drobak, Norway). Eighteen-day-old plants were subjected to heat stress, essentially as LeBlanc et al. (2018) described, to increase the efficiency of Cas9 activity. The heat stress protocol consisted of 4 cycles consisting of two phases each: 30 h heat stress at 37°C followed by 42 h recovery at 22°C. After the heat treatment, plants were exposed to normal growth conditions. Two rosette leaves of 40-day-old plants were collected, and genomic DNA was extracted with the Edwards extraction protocol (Edwards et al., 1991). The region around the expected mutation site was amplified by PCR using the primers P934 and P874 (Supplementary Table 1). The amplified PCR fragment was sequenced (P938, Supplementary Table 1) (LGC Genomics, Berlin, Germany). The resulting chromatograms were analyzed with BioEdit® software (https://bioedit.software.informer.com/7.2/). Sequences were aligned with wild-type sequences to detect mutations with Clustal Omega (Sievers et al., 2011).

T2 seeds were sown on MS plates and were transferred to artificial soil after 2 weeks. Once plants were 33 d old, two rosette leaves were collected to extract genomic DNA. PCR analysis was performed as described above. The presence of Cas9 was also tested by PCR analysis (L205 and L206 primers, Supplementary Table 1). In the T2 stage, the objective is to obtain plants with the mutation but no longer containing the Cas9 sequence. Cas9-free mutants T2 plants were selected and sent for sequencing. The results were analyzed using BioEdit®, Clustal Omega, and TIDE (Brinkman et al., 2014). The TIDE analysis allowed us to estimate the frequency of insertions and deletions (indels). If mutated candidate plants were considered heterozygous, they were submitted to a third selection.

In the T3 generation, five homozygous knockout lines were obtained: KO1 (1 bp deletion), KO2 (1 bp insertion), KO3 (1bp deletion), KO4 (1 bp deletion), and KO5 (1 bp insertion) (Supplementary Figure 2). More experimental details have been described by Dubiel et al. (2020).

### Phenotypic Analysis of *Arabidopsis thaliana* Lines

For the phenotypic characterization of the rosette area, 7-day-old seedlings grown *in vitro* on MS plates were transferred to 6-well plates (Greiner Bio One International). Plates were kept in a growth chamber set at 21°C with a 16/8 h light/dark photoperiod (200–400 μmol PAR m^−2^s^−1)^. Images of 17-d-old *Arabidopsis* rosettes from *Arabidopsis* lines (wild-type, overexpression, knockdown, knockdown lines, and *npr1-1* mutant) were taken and analyzed using a custom-built automated imaging system (PhenoVation Life Sciences, Wageningen, The Netherlands) as previously described by Tan et al. (2020).

### Infection Assays

Infection assays were performed using the flood inoculation technique, essentially as described by Ishiga et al. (2011) with some modifications. *Pseudomonas syringae* pv. *tomato* DC3000 (*Pst* DC3000) was kindly provided by Prof. Dr. Monica Höfte (Laboratory of Phytopathology, Ghent University, Belgium). *Pseudomonas syringae* pv. *tomato* DC3000 Δ*fliC* mutant (CUCPB5467) was kindly provided by Prof. Dr. Alan Collmer (School of Integrative Plant Science, Plant Pathology and Plant-Microbe Biology Section, Cornell University, USA). Bacteria were plated on King's B plates the week before infection. *Pst* DC3000 and *Pst* DC3000 Δ*fliC* mutant were grown in King's B containing 50 μg/mL of rifampicin. Liquid bacterial cultures were started using liquid King's B and were grown at 28°C at 200 rpm until the OD600 reached the logarithmic phase (OD600 = 0.6–1). At that point, bacterial cultures were centrifuged at 3,000 g for 5 min. Bacterial cells were re-suspended in 10 mM MgSO_4_ to reach OD600 = 0.015. The suspension was supplemented with 0.025% Silwet-77 [GE Specialty Materials (Suisse) S.a.r.l., Switzerland], a non-ionic organosilicon surfactant that facilitates the stomatal penetration of aqueous solutions (Zidack et al., 1992). The mock solution was composed of 10 mM MgSO_4_ and 0.025% Silwet-77.

Flood inoculation infections were performed using 14-day-old *Arabidopsis thaliana* seedlings grown *in vitro*. Seedlings grown on plates with MS medium were covered with a mock solution or bacterial suspension for 10 min. After inoculation, plates were kept in a controlled growth chamber set at 21°C with a 16/8 h light/dark photoperiod. At indicated time points post-infection, plant samples were collected for phenotypic characterization of the plants, quantification of leaf damage, quantification of GFP-labeled *Pst* DC3000 by determining the fluorescent signal and RNA extraction and qPCR analysis.

### Phenotypic Evaluation of the Effect of *Pst* DC3000 Infection on *Arabidopsis thaliana* Plants

Phenotypic analysis of infected and mock-treated *Arabidopsis* plants was performed using a custom-built automated imaging system to obtain high-resolution multispectral images (CropReporter, Phenovation Life Sciences, Wageningen, The Netherlands). Following traits were measured according to the manufacturer's specification: (i) projected leaf area, expressed in the number of pixels, (ii) maximum quantum efficiency of photosystem II (Fv/Fm) (Baker, 2008), (iii) the chlorophyll index (ChlIdx) (Gitelson et al., 2003), and (iv) the modified anthocyanin reflectance index (mARI) (Gitelson et al., 2009). The ChlIdx and mARI are correlated to the amount of chlorophyll and anthocyanins present in the leaf, respectively. Following formulas were used to calculate these indexes:

$$\begin{array}{lll} \text{ChlIdx} & = & \left(\text{ρ}\left(770\text{nm}\right)-\text{ρ}\left(710\text{nm}\right)\right)/\text{ρ}\left(710\text{nm}\right)\\ \text{mARI} & = & \text{ρ}770\times \left(1/\rho 550-1/\rho 710\right) \end{array}$$

More information about multispectral imaging and measured traits included in this study can be found in Meng et al. (2020).

### Quantification of Damaged Leaf Area After *Pseudomonas* Infection

*Arabidopsis thaliana* plants were kept under normal growth conditions after bacterial infection. Leaves were collected 4-days after *Pst* DC3000 or *Pst* DC3000 Δ*fliC* infection. Pictures were taken with a Leica DFC400 microscope (Leica, Heerbrugg, Germany). Images were analyzed using APS Assess 2.0 to quantify the lesion areas on a random set of individual leaves for each plant line. Leaf damage was presented as the percentage of damaged leaf area after bacterial infection.

### Quantification of Fluorescent *Pseudomonas* in Infected Plants

To measure differences between the bacterial population in *Arabidopsis thaliana* plant lines, seedlings were infected with a GFP-labeled *Pst* DC3000, kindly provided by Prof. Dr. Sheng Yang He (Howard Hughes Medical Institute, Michigan State University, USA), using the flood inoculation method described above. Images were taken with a custom-built automated imaging system (CropReporter, Phenovation Life Sciences, Wageningen, Netherlands) at 1, 3, and 5 days post-infection. The cGFP (corrected GFP) values reflect the presence of GFP-labeled *Pst* DC3000 in the plant and have been corrected for auto fluorescence of the plants.

### RNA Extraction, cDNA Synthesis, and RT-PCR Analysis

Aerial parts of *Arabidopsis* seedlings were collected at 1- and 3-days post-infection (dpi) and stored at −80°C. RNA was extracted using TriReagent (Sigma-Aldrich). To avoid the presence of any residual DNA, samples were submitted to DNAse treatment (Fermentas Kit, St. Leon-Rot, Germany). The concentration and purity of the RNA were measured with Nanodrop 200 (Thermo Scientific, Waltham, MA). cDNA was synthesized starting from 2 μg DNA-free RNA using M-MLV Reverse Transcriptase kit (Invitrogen), according to the instructions of the manufacturer. The final product was diluted 1:5 with RNA-free bidestilled water. The quality of the cDNA was tested by RT-PCR using specific qPCR primers for the SUMO-conjugating enzyme UBC9 gene (evd731 and evd732, Supplementary Table 2). The PCR amplification product was analyzed by gel electrophoresis on a 3% agarose gel in a 0.5% TAE buffer.

### Quantitative RT-PCR (qRT-PCR) Analysis

Quantitative RT-PCR analyses were performed using the 96-well CFX Connect™ Real-Time PCR Detection System (Bio-Rad Laboratories, Hercules, CA) with Bio-Rad IQ SYBR Green Supermix (Thermo Scientific, Waltham, MA). The reaction mix was composed of 1x SensiMix™ SYBR, 2 ng/μL first-strand cDNA, and 10 μM of gene-specific forward and reversed primers (Supplementary Table 2) in a total volume of 20 μL. The program was as follows: 10′ 95°C – 41 × (15″ 95°C – 25″ 60°C – 20″ 72°C) ending with generation of a melting curve (a gradual increase of temperature from 65 to 95°C rising by 0.5°C every 5 s). Bio-Rad CFX Manager 3.1 software was used to analyze the melting curves. The qBASE software (Hellemans et al., 2007) was used to check the stability of the reference genes PP2A, TIP41, and UBC9 (Czechowski et al., 2005). The results were statistically analyzed using the pairwise fixed reallocation randomization test using the REST-384 software (Corbett Research, Pfaffl et al., 2002). For each genotype, three independent biological replicates were analyzed with two technical replicates. The gene expression values were normalized using at least two reference genes.

### Statistical Analysis

Statistical analyses were performed using the software SPSS Statistics 26 (IBM, Armonk, NY, USA). Shapiro–Wilk test amended with a Bonferroni correction was used to check the assumption of normality, and Levene's test was used for the equality of variances. In multiple pairwise comparisons, ANOVAs were run, followed by Tukey *post-hoc* tests to identify the statistical differences between treatments. In pairwise comparisons, independent samples *T*-tests were run when data were normally distributed and Mann–Whitney *U*-test for not normally distributed data. All results are shown as mean ± SD.

## Results

### Transgenic Lines With Altered Levels of F-Box Nictaba

To unravel the importance of F-Box Nictaba in plant defense against *Pseudomonas* infection, a comparative analysis was made for a set of *Arabidopsis* lines with different levels of F-Box Nictaba expression. Overexpression lines (OE4 and OE6), as well as a knockdown line, were available from previous studies (Stefanowicz et al., 2016). Five knockout lines were generated in this study using the CRISPR technology (Supplementary Figures 1, 2). KO2 and KO5, both knockout lines with one bp insertion, were selected for detailed analysis. Seedlings from overexpression, knockdown, and knockout lines for F-Box Nictaba were subjected to flood inoculation experiments with bacterial suspensions from different *Pseudomonas* strains. Data for the transgenic lines and some control lines, particularly wild-type Columbia-0 (WT) plants and *npr1-1* lines deficient in the salicylic-acid-mediated systemic acquired resistance pathway, were included for comparison.

**Figure 1:**
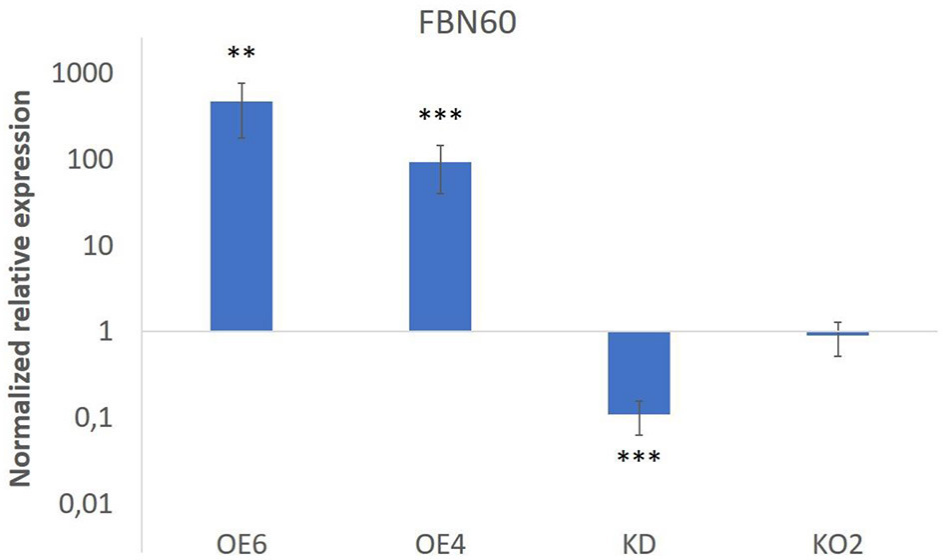
F-Box Nictaba transcript levels in transgenic lines. Results represent the fold change (and SE) for FBN60 transcripts in transgenic lines compared with WT plants. Asterisks indicate statistically significant differences between WT and transgenic lines (^**^p ≤ 0.01; ^***^p ≤ 0.001). WT, wild-type plants.

Transcript levels for F-Box Nictaba were quantified in 15-day-old seedlings (Figure 1). Overexpression lines OE4 and OE6 showed an increase in transcript levels for F-Box Nictaba up to 93-fold and 468-fold, respectively. In contrast, the knockdown line (KD) revealed a down-regulation in the level of F-Box Nictaba by 10-fold compared with wild-type plants. Transcript levels for F-Box Nictaba in the Knock-out line KO2 did not differ from wild-type plants. This could be due to primers not covering the area with the deletion, but the RNA transcript will generate a defective protein.

### *Pst* DC3000 Infection Causes a Reduction in Rosette Size, in the Efficiency of Photosystem II and Chlorophyll Content

**Figure 2:**
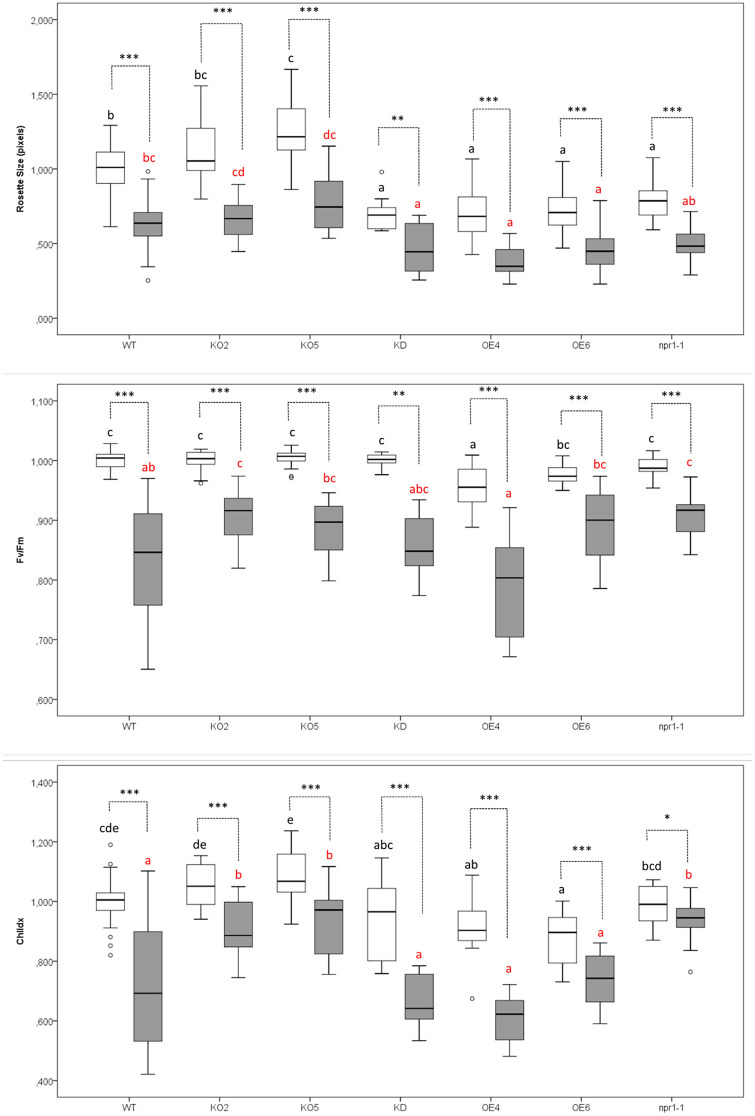
Effect of *Pst* DC3000 infection on rosette size, the efficiency of photosystem II (Fv/Fm), and chlorophyll content (ChlIdx) in 17-day-old *Arabidopsis* plants at 3 days after inoculation compared with mock-treated plants. Graphs show mean values with error bars representing standard deviation. For this graph, values of all plant lines and treatments were normalized to the WT mock of each experiment, allowing us to represent two different experiments together in one graph. White boxes represent Mock-treated samples and gray boxes *Pst* DC3000 infected samples. Comparison of Arabidopsis lines within mock-treated plants (black letters) or *Pst* DC3000 infected plants (red letters) was done with a one-way ANOVA analysis followed by a *post-hoc* Tukey test. Significant letters indicate differences at a level of *p* < 0.05. The comparison between mock and *Pst* DC3000 treated plants within a plant genotype was done using independent samples *T*-test for normally distributed data and Mann-Whitney test for non-normally distributed samples. Asterisks indicate statistically significant differences between treatments within the same transgenic line (^*^p ≤ 0.1; ^**^p ≤ 0.05; ^***^p ≤ 0.001). Number of observations (individual rosettes) in mock treatment: WT = 36, KO2 = 18, KO5 = 18, KD = 10 OE4 = 12, OE6 = 18, *npr1-1* = 18. Number of observations (individual rosettes) in *Pst* DC3000 infection treatment: WT = 48, KO2 = 18, KO5 = 18, KD = 10, OE4 = 12, OE6 = 24, *npr1-1* = 17.

**Figure 3:**
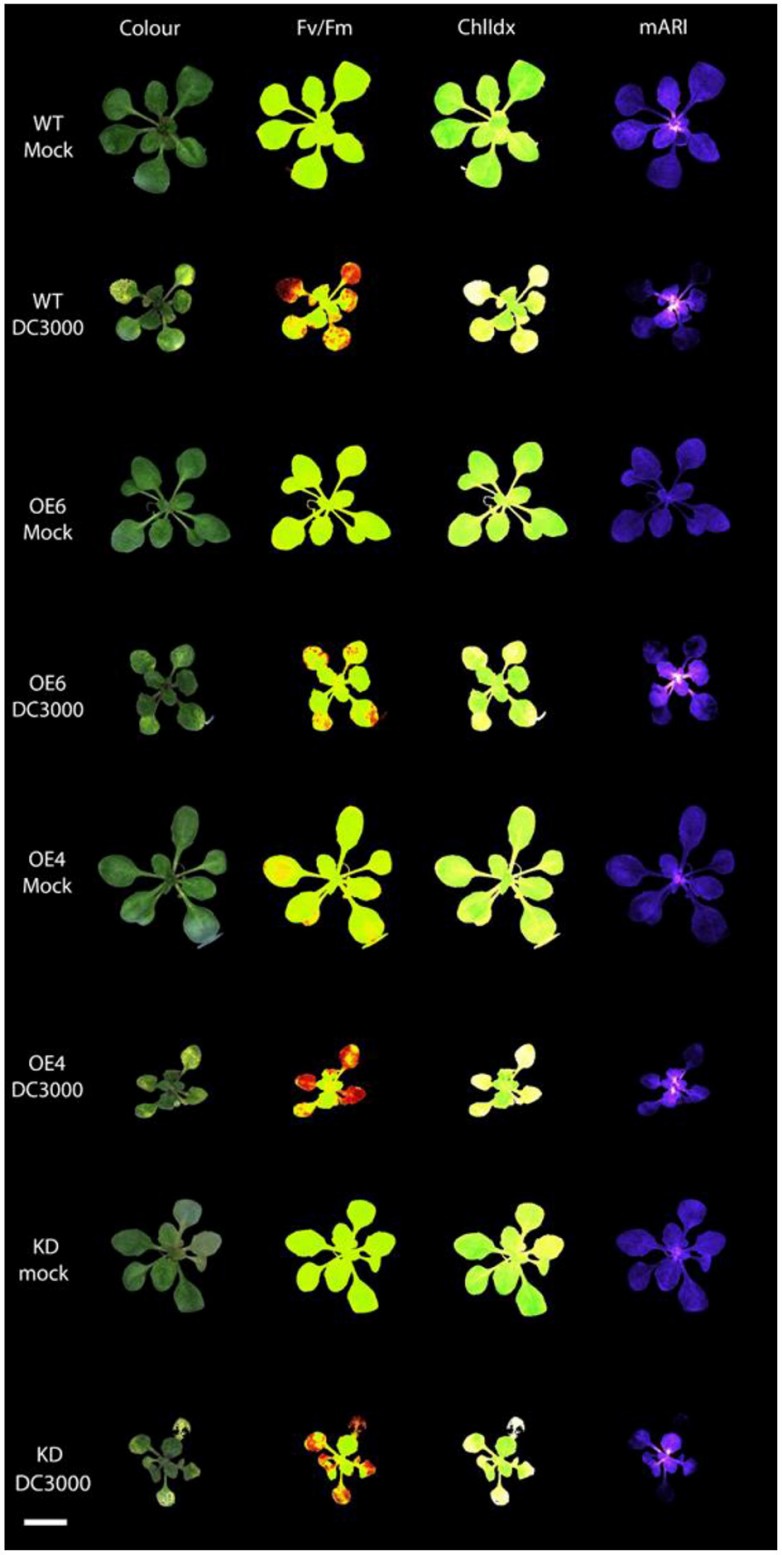
Multispectral images of *Arabidopsis* rosettes at 3 days after mock treatment and *Pst* DC3000 inoculation. The different images represent the RGB Color image; Fv/Fm: efficiency of photosystem II; ChlIdx: chlorophyll index, a measure for chlorophyll; mARI: modified anthocyanin reflectance index, a measure for the amount of anthocyanin content. Scale bar represents 1 cm.

**Figure 4:**
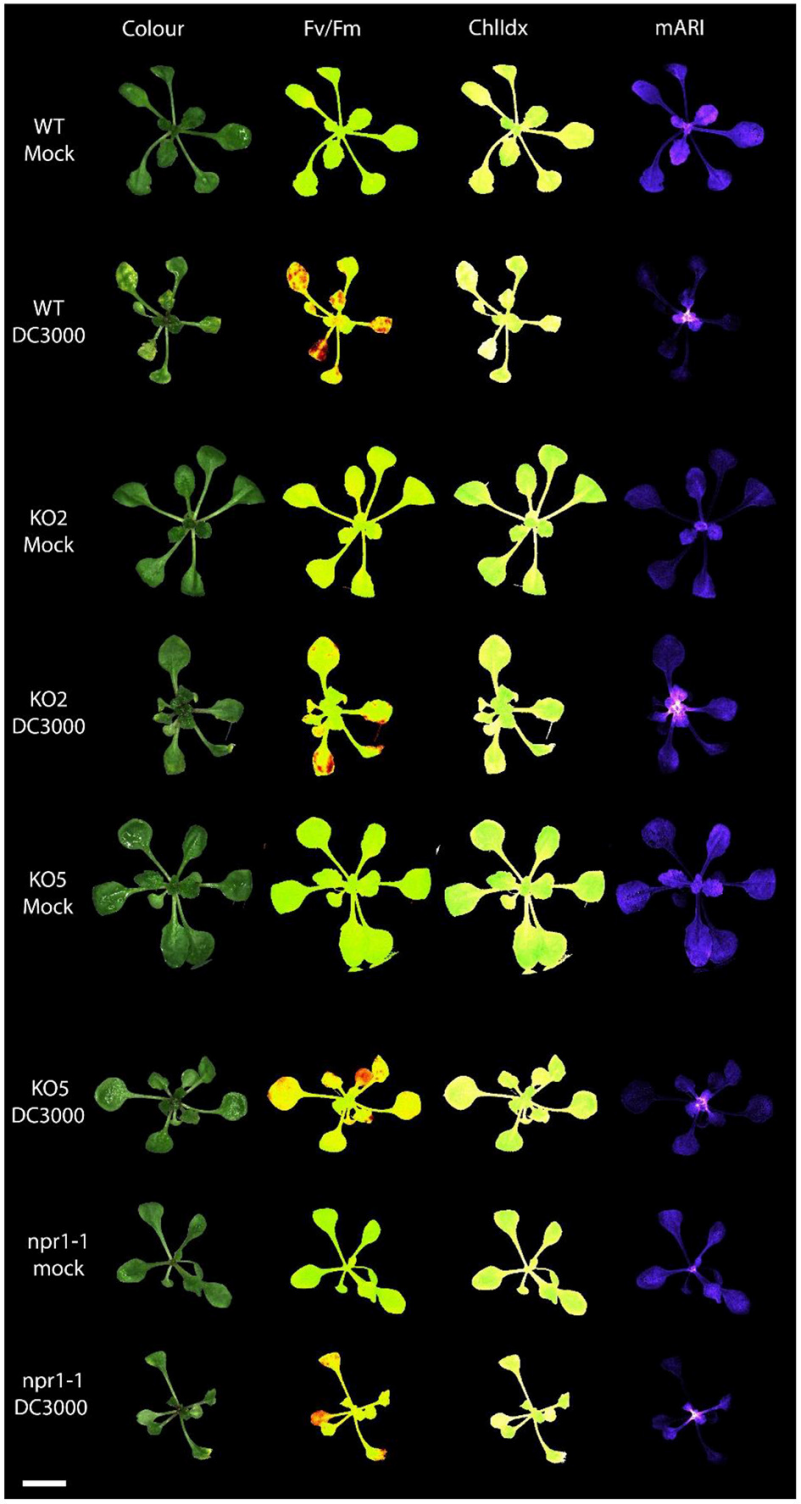
Multispectral images of *Arabidopsis* rosettes 3 days after mock treatment and *Pst* DC3000 inoculation. The different images represent the rosette measuring the following traits: RGB Color image; Fv/Fm: efficiency of photosystem II; ChlIdx: chlorophyll index, a measure for the amount of chlorophyll; mARI: modified anthocyanin reflectance index, a measure for the amount of anthocyanin content. Scale bar represents 1 cm.

Fourteen-day-old *Arabidopsis thaliana* plants were infected with *Pst* DC3000 and analyzed at 3 days after inoculation to establish the effect of bacterial infection on three different plant parameters: rosette size, the efficiency of photosystem II and chlorophyll content (Figures 2–4).

Rosettes of plants overexpressing F-Box Nictaba were significantly smaller than rosettes from wild-type plants grown under identical conditions (Figure 2). KD and *npr1-1* plants presented the same trend. In contrast, the knockout lines KO2 and KO5 yielded larger rosettes. When plants were infected with *Pst* DC3000, rosette sizes of all the plant lines under study were significantly reduced as a symptom of the infection. After bacterial infection, the rosettes of OE4, OE6, and KD plants were still significantly smaller than the rosettes of WT plants.

The efficiency of photosystem II (Fv/Fm) was compared for mock-treated plants and *Pst* DC3000 infected plants. The non-infected wild-type plants showed a significantly higher efficiency of photosystem II than the F-Box Nictaba overexpressing line OE4. After infection with *Pst* DC3000, KO2 and *npr1-1* plants were the only lines showing a significantly higher efficiency of photosystem II than wild-type infected plants.

Mock-treated OE6 and OE4 showed a lower chlorophyll index than mock-treated wild-type plants. However, after infection, F-Box Nictaba overexpressing lines are not significantly different from wild-type plants anymore, indicating that they perform better against bacterial infection. However, the chlorophyll index in KO2, KO5, and *npr1-1* plants were significantly higher than in wild-type plants after infection.

### F-Box Nictaba Overexpression Line Shows Reduced Leaf Damage After *Pseudomonas* Infection

**Figure 5:**
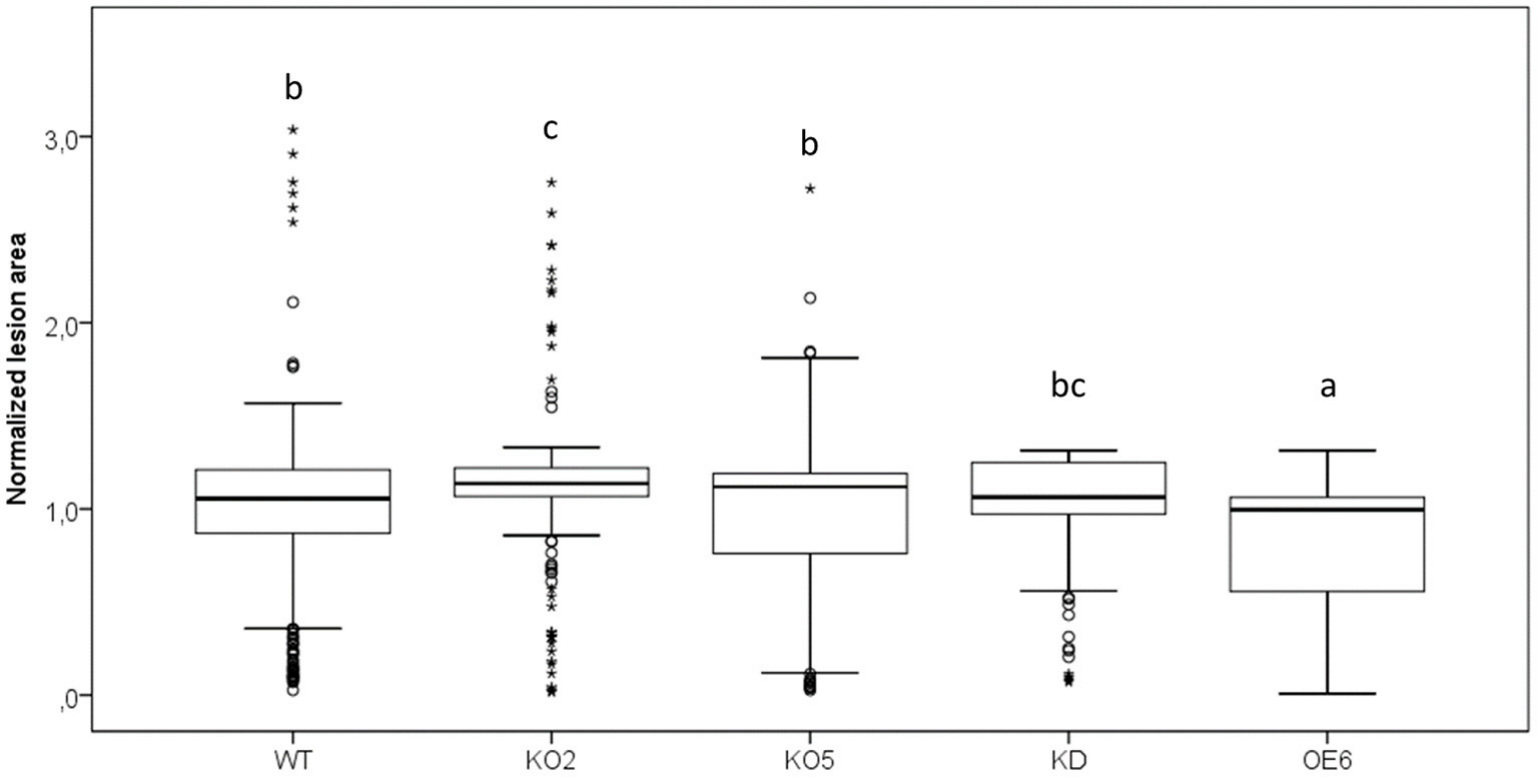
Quantification of damaged leaf area after *Pst* DC3000 infection in WT, KO2, KO5, OE6, and KD plants. Leaf damage was measured as chlorotic lesion area in total leaf area and normalized to the WT lesion area. These results are based on three independent biological replicates. For this graph, values of all plant lines were normalized to the WT of each experiment, allowing us to represent two different experiments together in one graph. Comparison of Arabidopsis lines was done with a one-way ANOVA analysis followed by a *post hoc* Tukey test. Significant letters indicate differences at a level of *p* < 0.05.

Wild-type plants and transgenic lines with different levels of F-Box Nictaba expression were subjected to *Pst* DC3000 infection, and the chlorotic lesions representing damaged leaf area as a result of *Pst* DC3000 colonization in the apoplast were quantified (Figure 5). Leaves of the OE6 line clearly showed a reduced level of damaged leaf area compared with wild-type plants, whereas there was no difference in damaged leaf area between knockdown and wild-type plants. The knockout line KO2 showed a higher lesion area than wild-type plants, but the results for line KO5 did not show any difference with wild-type plants.

### F-Box Nictaba Overexpression Lines Show Less Bacterial Growth After *Pseudomonas* Infection

**Figure 6:**
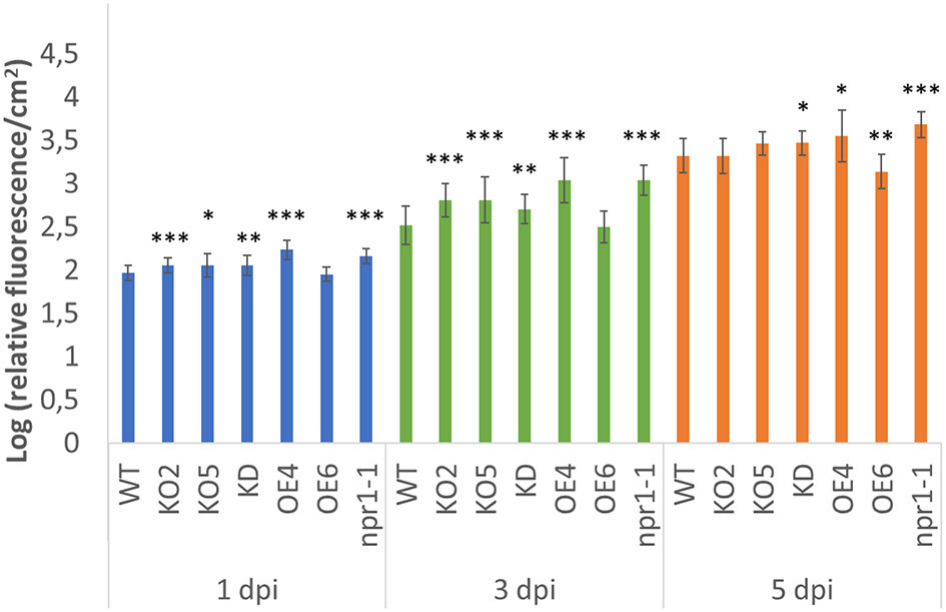
Fluorescence measured in 2-week-old *Arabidopsis thaliana* plants infected with GFP-labeled *Pst* DC3000 strain at 1-, 3-, and 5-days post-infection. Fluorescence was calculated using the log of the ratio cGFP/cm^2^ in order to represent the fluorescence in the plant. Comparison between wild-type plants and transgenic lines was performed using independent samples *T*-test for normally distributed data and Mann-Whitney test for non-normally distributed. Asterisks indicate statistically significant differences between WT and transgenic lines (^*^p ≤ 0.05; ^**^p ≤ 0.01; ^***^p ≤ 0.001). cGFP: corrected Green Fluorescent Protein fluorescence.

Two-week-old *Arabidopsis* seedlings were inoculated with a GFP-labeled *Pst* DC3000 strain. Previous studies reported the correlation between fluorescence intensity and bacterial population (Wang et al., 2007; Pasin et al., 2014; Wilson et al., 2018; Hupp et al., 2019), showing that fluorescence measurements allow estimating the bacterial population present in the leaves, offering the additional benefit of non-destructive monitoring of the bacterial population. The growth of the *Pseudomonas* bacteria was quantified by measuring the fluorescence in the plants at different time points after infection (Figure 6). In general, fluorescence levels in the plants increased from day 1 to day 5. The mutant *npr1-1* plants showed a clear increase in fluorescence compared with wild-type plants over time. With respect to all FBN60 mutants, all the plant lines, except OE6 plantlets, showed significantly higher fluorescence than wild-type plants at 1 dpi and 3 dpi. At 5 dpi, the FBN60 overexpressing line OE6 showed significantly less fluorescence than wild-type plants. However, FBN60 overexpressing line OE4 showed significantly higher fluorescence than wild-type plants at 5 dpi. KO2, KO5, and KD plants showed a slightly higher fluorescence than wild-type plants at 1 and 3 dpi. However, both KO lines were not significantly different from wild-type plants at 5 dpi.

### Plants Overexpressing F-Box Nictaba Show Increased mARI Values After *Pst* DC3000 Infection

**Figure 7:**
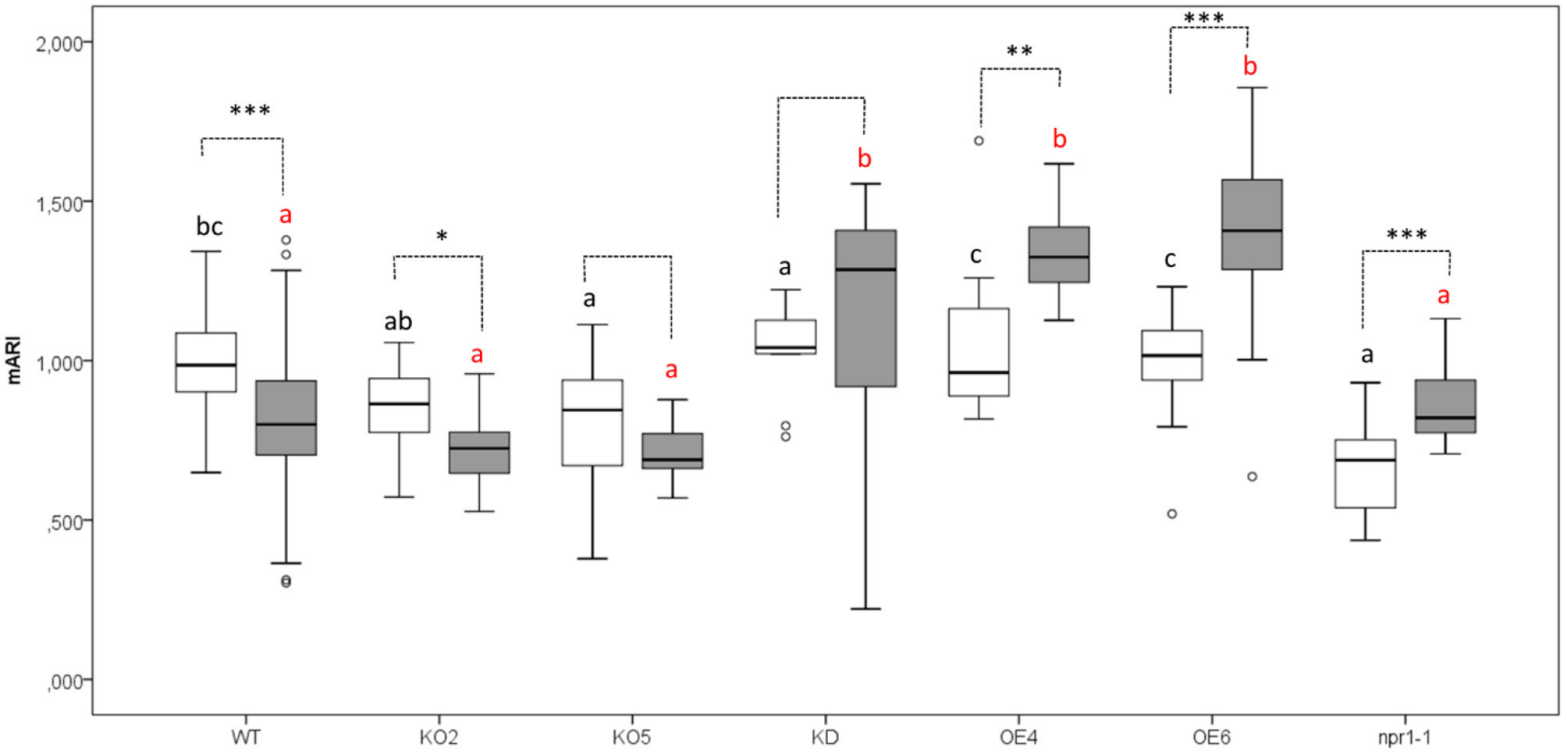
Anthocyanin content (AriIdx) in 17-day-old *Arabidopsis* plants at 3 days after *Pst* DC3000 inoculation. Graphs show mean values with error bars representing standard deviation. For this graph, values of all plant lines and treatments were normalized to the WT Mock of each experiment, allowing us to represent two different experiments together in one graph. White boxes represent mock-treated samples and gray boxes *Pst* DC3000 infected samples. Comparison of Arabidopsis lines within *Pst* DC3000 treated plants (red letters), or mock-infected plants (black letters) was done with a one-way ANOVA analysis followed by a *post-hoc* Tukey test. Significant letters indicate differences at a level of *p* < 0.05. The comparison between mock and *Pst* DC3000 infected plants within a plant genotype was done using independent samples *T*-test for normally distributed data and Mann-Whitney test for non-normally distributed samples. Asterisks indicate statistically significant differences between treatments within the same transgenic line (^*^p ≤ 0.1; ^**^p ≤ 0.05; ^***^p ≤ 0.001). Number of observations (individual rosettes) in mock treatment: WT = 36, OE6 = 18, OE4 = 12, KD = 10, KO2 = 18, KO5 = 18, *npr1-1* = 18. Number of observations (individual rosettes) in *Pst* DC3000 infection treatment: WT = 48, OE6 = 24, OE4 = 12, KD = 10, KO2 = 18, KO5 = 18, *npr1-1* = 17.

The anthocyanin content was analyzed in mock-treated, and *Pst* DC3000 infected plants (Figures 3, 4). A comparative analysis between wild-type and transgenic plants revealed that KO5, KD, and *npr1-1* showed lower mARI levels than mock-treated wild-type plants. Moreover, overexpression lines showed a higher mARI value than KD, KO2, KO5, and *npr1-1* plants in the same conditions. After *Pst* DC3000 infection, both OE4 and OE6 lines showed an increased mARI level, while it was reduced in wild-type and KO2 plants. KD plants showed a significantly lower mARI level in the mock plants, but similar to OE lines the anthocyanin levels were significantly higher after infection than wild-type plants. However, the infection did not increase the anthocyanin content in KD plants. In the same line, KO lines revealed a lower anthocyanin content than wild-type plants after mock treatment, but these KO lines showed no major changes in mARI levels after infection. Although the KO2 line showed a small but significant reduction after infection, the KO5 line did not yield any change in anthocyanin levels. Like the overexpression lines, the mutant *npr1-1* plants revealed an increased anthocyanin content after *Pst* DC3000 infection. Mock treated *npr1-1* plants had significantly lower mARI values than wild-type plants, but after *Pst* DC3000 infection, these lines showed similar results (Figure 7).

### *Pst* DC3000 Δ*fliC* Mutant Causes Reduced Infection Symptoms in *Arabidopsis thaliana* Plants

**Figure 8:**
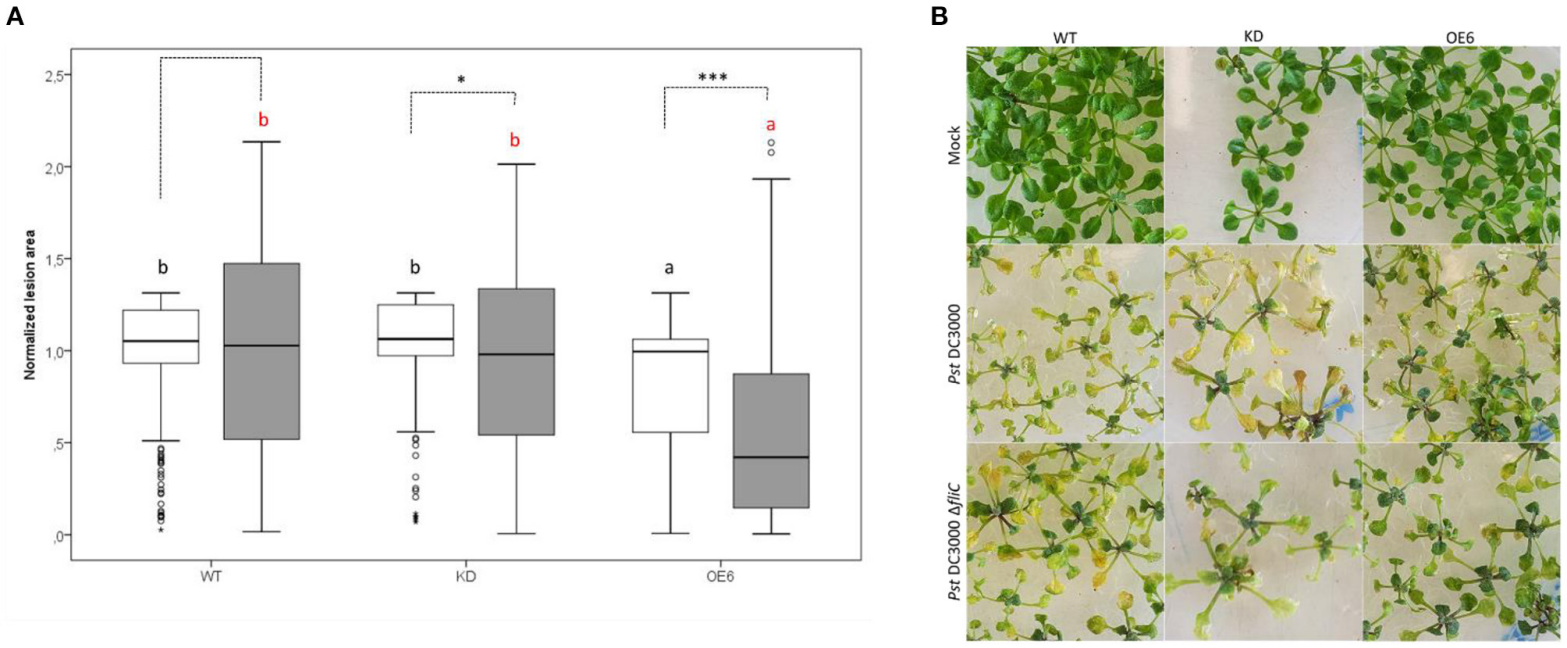
Damaged leaf area after *Pst* DC3000 Δ*fliC* mutant infection in WT, OE6, and KD plants and comparison with *Pst* DC3000 strain. Quantification of lesion area **(A)**. The leaf damage was measured as chlorotic lesion area in total leaf area and normalized to the WT lesion area. These results are based on three independent biological replicates. For this graph, values of all plant lines and treatments were normalized to the WT of each experiment, allowing us to represent two different experiments together in one graph. White boxes represent *Pst* DC3000 infected plants and gray boxes Δ*fliC*-infected plants. Comparison of Arabidopsis lines within *Pst* DC3000 treated plants (black letters) or Δ*fliC* infected plants (red letters) was done with a one-way ANOVA analysis followed by a *post hoc* Tukey test. Significant letters indicate differences at a level of *p* < 0.05. Comparing mock and *Pst* DC3000 infected plants within a plant genotype was done using independent samples *T*-test for normally distributed data and Mann–Whitney test for non-normally distributed samples. Asterisks indicate statistically significant differences between treatments within the same transgenic line (^*^p ≤ 0.1; ^***^p ≤ 0.001). Phenotype of Arabidopsis lines after mock treatment and infection with *Pst* DC3000 and Δ*fliC* mutant **(B)**.

The virulence of the *Pst* DC3000 mutant strain, deficient in flagellin Δ*fliC*, was analyzed on WT, OE6, and KD plants, and the damaged leaf areas were compared with the damage caused by *Pst* DC3000 (Figure 8). As shown in Figure 8B, the *Pst* DC3000 Δ*fliC* mutant caused less lesion area than *Pst* DC3000. Quantitative analysis revealed that the mutant strain was less virulent on OE6 plants. These F-Box Nictaba overexpressing plants showed a significantly reduced level of damaged leaf area than wild-type plants. However, infected knockdown plants did not show any significant differences compared with infected wild-type plants. The virulence of the *Pst* DC3000 mutant strain seems to be reduced compared with *Pst* DC3000, at least for OE6 plants. No statistical differences were found between *Pst* DC3000-infected and Δ*fliC*-infected wild-type plants.

### Overexpression of F-Box Nictaba Leads to Higher Up-Regulation of SA-Related Defense Genes After *Pseudomonas* Infection

**Figure 9:**
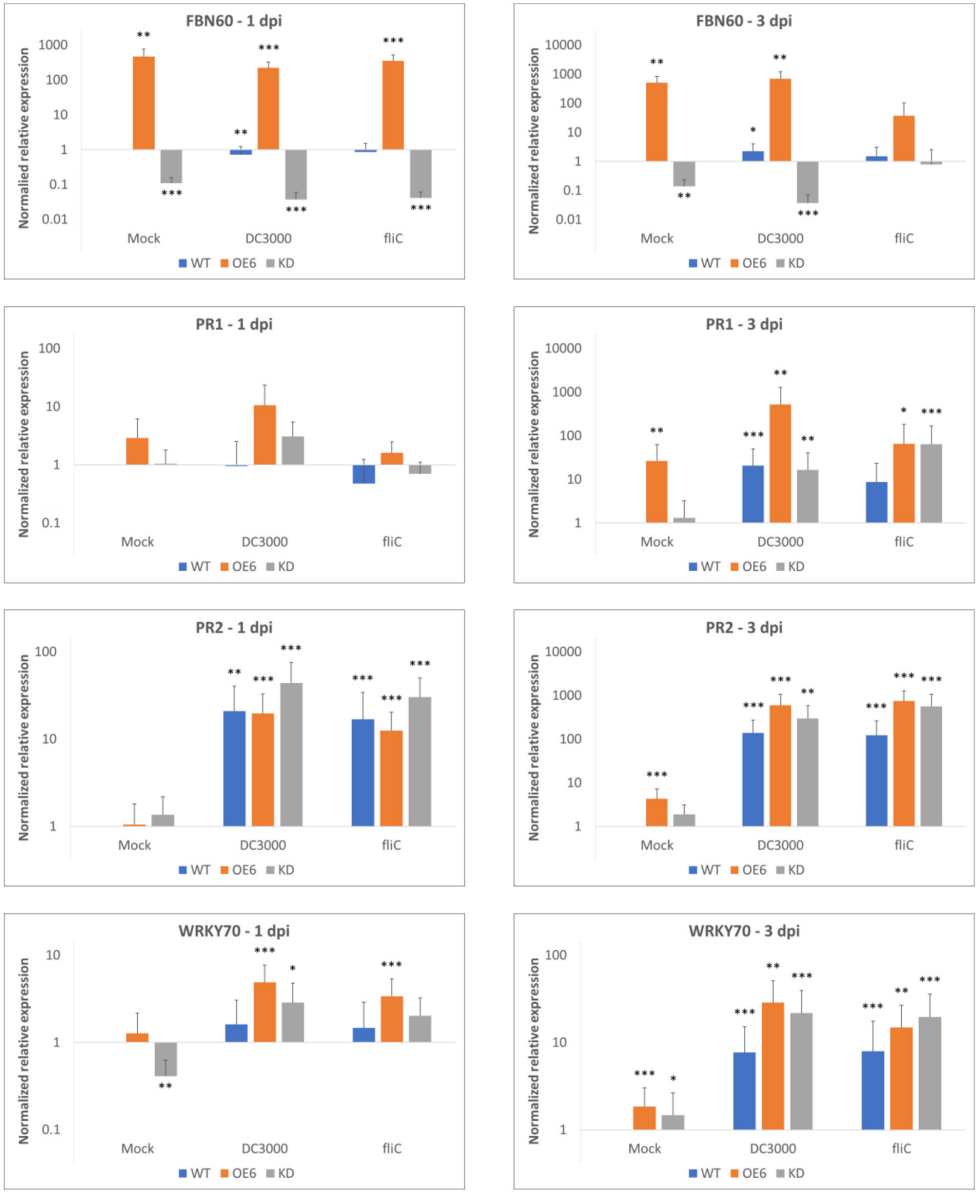
Relative transcript levels for F-Box Nictaba (FBN60, *At2g02360*) and SA-defense pathway-related genes WRKY70 (*At3g56400*), PR1 (*At2g14610*), and PR2 (*At3g57260*) in *Arabidopsis thaliana* plants after inoculation with *Pst* DC3000 or *Pst* DC3000 Δ*fliC* mutant. Results were determined by qPCR analysis for three independent biological replicates. Error bars represent standard errors. Asterisks indicate the significance of statistical differences of the samples compared with wild-type mock-treated plants (^*^p < 0.05; ^**^p < 0.01; ^***^p < 0.001). Graphs are shown on a logarithmic scale.

Transcript levels for *FBN60* were quantified in 2-week-old plants at 1 and 3 days after inoculation with *Pst* DC3000 and its mutant *Pst* DC3000 Δ*fliC* (Figure 9). In the mock treatment, transcripts for OE6 *FBN60* were significantly upregulated 468-fold and 514-fold at 1 and 3 dpi, respectively. In contrast, after mock inoculation in the KD line, *FBN60* was significantly downregulated (9-fold, and 7-fold at 1 and 3 dpi, respectively) compared with the wild-type plants.

After inoculation with *Pst* DC3000, wild-type plants had a slight down-regulation of *FBN60* at 1 dpi. However, at 3 dpi, F-Box Nictaba was 2-fold upregulated. However, the expression of *FBN60* in OE6 did not differ significantly between the mock treatment and *Pst* DC3000. After inoculation with the Δ*fliC* mutant, expression of *FBN60* was not significantly different from the wild-type mock-treated plants at 3 dpi in OE6 plants.

To confirm which defense-related pathways are activated following infection, the expression of genes related to SA-dependent defense pathways were studied after *Pseudomonas* infection. At 3 dpi with both *Pseudomonas* strains, all three genes, *PR1, PR2*, and *WRKY70*, were significantly upregulated. Overexpression (OE6) plants showed a higher expression of *PR1* (25-fold upregulation) and *WRKY70* (3-fold upregulation) compared with wild-type plants (*P*-value: 0.0015) and a higher expression of *PR1* (31-fold upregulation) compared with KD plants (*P*-value: 0.001) after *Pst* DC3000 infection. However, no difference was found when the wild-type and OE6 plants were compared to express disease-related genes at 3 dpi after inoculation with *Pst* DC3000 Δ*fliC* mutant (results not shown).

The expression of *PR1* in wild-type, OE6, and KD plants was significantly upregulated after *Pst* DC3000 treatment compared with wild-type mock-treated plants at 3 dpi, whereas *PR2* was already significantly upregulated at 1 dpi for both bacterial strains. Interestingly, OE6 plants showed constitutive induction of *PR1, PR2*, and *WRKY70* at 3 dpi when they are mock-treated.

## Discussion

To explore the role of F-Box Nictaba (*At2g02360*) in the plant defense of *Arabidopsis thaliana* against bacterial infection, 2-week-old plants were infected with *Pseudomonas syringae* using the flood inoculation technique (Ishiga et al., 2011). Transcript levels for *At2g02360* were 2-fold upregulated in 2-week-old wild-type *Arabidopsis* plants at 3 days after *Pst* DC3000 infection. These data are similar to the results described for 5-week-old *Arabidopsis* plants infected by spraying with a bacterial solution of *Pst* DC3000 (Stefanowicz et al., 2016). All these data support the idea that F-Box Nictaba is a defense-related protein.

The phenotypic characterization of *Arabidopsis* plants after *Pst* DC3000 infection provided us with interesting information regarding the plant status of the different transgenic lines. OE6 plants showed reduced lesion area compared with wild-type plants after *Pst* DC3000 infection. However, there were no significant differences between wild-type plants and neither OE6 nor OE4 regarding the efficiency of photosystem II (Fv/Fm) and chlorophyll index, a proxy for chlorophyll content. In contrast, when plants were mock-inoculated, both overexpression lines showed lower efficiency of photosystem II (Fv/Fm) and chlorophyll index values than wild-type plants, which is probably related to the energy cost of overexpressing F-Box Nictaba. A comparative analysis between both overexpression lines revealed that line OE6 behaves better than line OE4 after *Pst* DC3000 infection, related to the higher level of overexpression of FBN60 in OE6 compared with OE4.

To unravel the importance of F-Box Nictaba in the plant during its interaction with *Pseudomonas syringae*, the same experiment was performed for F-Box Nictaba knockdown and knockout lines. In contrast to the overexpression plants, knockdown plants did not show differences in lesion area on leaves compared with wild-type plants. The same trend was followed in knockout line KO5, whereas the lesion area in KO2 was slightly higher than in wild-type leaves. Although the results with the OE lines clearly show that this protein takes part in the defense response against *Pst* DC3000, the data with the knockout lines suggest that F-Box Nictaba does not play an essential role in pathogen defense. We hypothesize that these data are the result of gene redundancy. At present, FBN60 is the only F-Box Nictaba gene studied in detail for gene regulation in response to pathogen infection. However, about 19 F-Box Nictaba related genes have been retrieved from the *Arabidopsis* genome. Therefore, it is likely that other F-Box Nictaba homologs could take over the role of *At2g02360* when this gene is no longer functional. Another possible explanation is that the F-Box Nictaba level in normal conditions is low in wild-type *Arabidopsis thaliana* plants. Transcript levels are upregulated after *Pst* DC3000 infection, but this increase in the transcript level is only a 2-fold change in wild-type plants treated with *Pst* DC3000. Consequently, the fact that the protein is no longer present in the knockout lines would not make an important difference.

The infection experiment with fluorescent bacteria supports the hypothesis that F-Box Nictaba plays a role in defense in *Arabidopsis thaliana* plants during *Pst* DC3000 infection. Judging from the fluorescence levels, the bacterial content in the plants increased over time. However, plants overexpressing F-Box Nictaba presented less bacterial colonization in the late stages of pathogenesis than wild-type plants, as demonstrated in a previous study (Stefanowicz et al., 2016). In the FBN60 overexpressing line OE6, no difference in fluorescence with wild-type plants was observed until 5 dpi, when OE6 plants showed reduced fluorescence values compared with wild-type plants. In the case of the overexpressing line OE4, higher fluorescence values were observed at 1, 3, and 5 dpi compared with wild-type plants. As previously proposed for infection symptoms, differences between both overexpression lines could be due to the different levels of protein overexpression, being much higher in the OE6 plants than in OE4 plants. In general, knockout and knockdown plants represented slightly higher fluorescence values than wild-type plants at 1 and 3 dpi, but at 5 dpi KO plants did not show differences with wild-type plants. Our data indicate that plant lines that lack F-Box Nictaba expression or contain reduced levels of F-Box Nictaba show infection symptoms comparable with wild-type plants. However, the overexpression of F-Box Nictaba leads to a better plant performance after *Pst* DC3000 infection, especially for OE6 plants that have a 400-fold increase in FBN60 levels. These data suggest that F-Box Nictaba is related to the plant defense response, possibly involved in a defense-response network detecting certain *Pseudomonas* effector(s), but it may not directly interact with the bacteria. The fact that bacteria live and multiply in the apoplastic space between cells, where effectors are injected, and F-Box Nictaba is located in the nucleus, and the cytoplasm (Stefanowicz et al., 2016) would support the latter idea.

Flagellin and its glycosylation structures are an important factor determining the virulence and host specificity of pathogens (Ishiga et al., 2005, Takeuchi et al., 2007). Six serine residues have been identified as common sites for glycosylation in the flagellin of *Pseudomonas syringae* pv. *tabaci* and *Pseudomonas syringae* pv. *glycinea* (Taguchi et al., 2006). The glycan attached to one of these serine residues was identified as a unique trisaccharide consisting of two rhamnosyl (Rha) residues and one modified 4-amino-4,6-dideoxyglucosyl residue, commonly known as viosamine. The ratio L-Rha*p* / D-Rha*p* was proven to be different between both pathovars (Takeuchi et al., 2007). Although little is known about the glycosylation profile for *Pst* DC3000, a comparative study exposed that *Pst* DC3000 had six glycans, identical to the ones in *Pta* 6605 (Yamamoto et al., 2011; Chiku et al., 2013). Plants and animals need to recognize bacterial flagellin to trigger defense responses. For example, *Nicotiana benthamiana* cells secrete the enzyme β-galactosidase 1, which targets terminally modified viosamine that is part of the flagellin O-glycan on *Pseudomonas syringae* pathovars (Buscaill et al., 2019). As such, these glycan structures on flagella are important for host immunity to bacterial pathogens. In addition to flagellin, pillin structures can also be glycosylated and are required for bacterial motility and promote bacterial virulence (Nothaft and Szymanski, 2010; Nguyen et al., 2012).

This study also performed experiments with the mutant *Pst* DC3000 Δ*fliC*, which does not present flagellin units. Consequently, flagella will be defective, and the possible glycosylation motifs expected on flagellin will no longer be present. Infection experiments showed that Δ*fliC*-infected plants presented less damaged area in leaves compared with *Pst* DC3000 infected plants. From our data, it is clear that the virulence of the *Pst* DC3000 Δ*fliC* mutant is lower than that of *Pst* DC3000. The lack of proper flagella reduces the motility of the bacterial cells (Kvitko et al., 2009; Nogales et al., 2015). Even if the flagella of *Pst* DC3000 presented a glycan structure that could be recognized by F-Box Nictaba, an interaction is unlikely since F-Box Nictaba is present in the nucleus and cytoplasm of the cell. It is not a membrane protein that could act as a pattern recognition receptor, as described for some lectin receptor-like kinases (Bellande et al., 2017). Consequently, the chance that F-Box Nictaba and *Pst* DC3000 that colonizes the apoplast will interact is low unless F-Box Nictaba can follow an unconventional route for secretion.

Owing to the presence of pattern recognition receptors on the plasma membrane, *Arabidopsis* can detect the presence of pathogens by recognizing pathogen- or damage-associated molecular patterns (PAMPs and DAMPs). This interaction is followed by the first defense responses against pathogen intrusion, such as the production of reactive oxygen species (ROS), modifications in the cell wall, synthesis of antimicrobial compounds, and expression of pathogen-related proteins (Gimenez-Ibanez and Rathjen, 2010; Trinh et al., 2018). Plant defense against *Pst* DC3000 is mainly regulated by SA-dependent signaling, which *Pst* DC3000 attempts to negate by producing the jasmonic acid (JA) analog coronatine, which suppresses SA-related signaling (Brooks et al., 2005; Spoel and Dong, 2008).

Our experiments indicate that F-Box Nictaba is related to resistance against *Pst* DC3000 by interacting with SA-dependent defense. Firstly, OE6 exhibited increased expression of the SA-related defense genes *PR1, PR2*, and *WRKY70* compared with wild-type plants after mock treatment, known to be involved in defense against *Pst* DC3000. Secondly, overexpression lines OE6 and OE4 showed a higher increase in mARI values, a proxy for anthocyanin content following infection with *Pst* DC3000 compared with wild-type plants, whereas knockout lines did not differ from the wild-type plants.

Previously it was demonstrated that the infection of *Pst* DC3000 in *Arabidopsis thaliana* plants causes the up-regulation of genes in response to the contact with hrp-regulated virulence factors, such as coronatine. Some of these genes encode enzymes related to anthocyanin biosynthesis, such as CHS (*At5g13930*), a putative anthocyanidin synthase (*At2g38240*), and UDP- anthocyanidin transferase (*At4g27570*) (Thilmony et al., 2006). It has also been proven that, under abiotic stress conditions, ROS can lead to anthocyanin accumulation since these compounds are produced as antioxidants to protect the plant itself against oxidative stress. This accumulation is produced by means of up-regulation of late genes involved in anthocyanin biosynthesis (like *TT3* and *TT18*) and their regulatory genes (for example, *PAP1, TT8, MYB113*, and *MYB114*). This accumulation helps maintain chlorophyll (Chl) and photosynthetic capacity (Xu et al., 2017).

We primarily found increased mARI levels in the youngest leaves, whereas a decrease in mARI was demonstrated in the older leaves. But we did not see a differential response of gene expression between the OE6 and KD lines following infection with the Δ*fliC* mutant. The Δ*fliC* mutant does not contain the PAMP flg22, a known inducer of SA-related defense in *Arabidopsis* (Tsuda et al., 2008). Together, with the study of Stefanowicz et al. (2016), in which FBN60 was induced following exogenous SA application, our results support a role for FBN60 as a mediator of resistance by increasing SA-related defense.

F-Box Nictaba has been demonstrated to be present mostly in the trichomes of young leaves of *Arabidopsis* plants (Stefanowicz et al., 2016). MYB-bHLH-WD40 (MBW) complexes regulate different cellular pathways in *Arabidopsis* (such as trichome initiation) and other plants. The MBW complex that regulates anthocyanin biosynthesis consists of the MYB transcription factors PAP1, PAP2, MYB113, and MYB114, the bHLH transcription factors TT8, GL3, and EGL3, and the WD40-repeat protein TTG1. This anthocyanin-regulatory complex and the MBW complex that controls trichome initiation named GL1/MYB82-GL3/EGL3-TTG1 present the same WD40 factor (and transcription factors) (Gou et al., 2011; Liang et al., 2014; Xie et al., 2016). The possible link between F-Box Nictaba and the process that regulates anthocyanin biosynthesis requires further investigation.

Our work reinforces the importance of the lectin F-Box Nictaba in the response of *Arabidopsis thaliana* plants against *Pseudomonas* infection. At present, it remains unclear whether lectin-carbohydrate interactions play a role in the plant response to *Pseudomonas* infection. Our findings clearly show that the overexpression of F-Box Nictaba helps the plant cope with a bacterial infection. Concomitantly a significant increase in the anthocyanin content was observed in the overexpression lines after bacterial infection. Future experiments should focus on the role of oxidative stress in F-Box Nictaba related plant responses. Although the detailed mechanism of how F-Box Nictaba triggers the plant immune response remains unsolved, our data contribute to better insights on the role of lectins in plant responses directed against *Pseudomonas* infection.

## Data Availability Statement

The raw data supporting the conclusions of this article will be made available by the authors, without undue reservation.

## Author Contributions

AR-P, EJMVD, KA, and MA designed the research. AR-P performed the experiments. EJMVD, KA, and MA supervised the experiments. AR-P, MA, and KA analyzed the data. AR-P, MA, and EJMVD wrote the manuscript. KA revised the manuscript. All authors have contributed, read, revised, and approved the submitted manuscript.

## Conflict of Interest

The authors declare that the research was conducted in the absence of any commercial or financial relationships that could be construed as a potential conflict of interest.

## Publisher's Note

All claims expressed in this article are solely those of the authors and do not necessarily represent those of their affiliated organizations, or those of the publisher, the editors and the reviewers. Any product that may be evaluated in this article, or claim that may be made by its manufacturer, is not guaranteed or endorsed by the publisher.
