# Supplementary material for: Overexpression of F-Box Nictaba Promotes Defense and Anthocyanin Accumulation in Arabidopsis thaliana After Pseudomonas syringae Infection
Source: Front Plant Sci. 2021 Jul 29;12:692606. doi: 10.3389/fpls.2021.692606 (PMC8358183; doi:10.3389/fpls.2021.692606)
Supplement: Supplementary file 1 [file Data_Sheet_1.PDF]

Supplementary Material

Overexpression of F-Box Nictaba promotes anthocyanin accumulation in *Arabidopsis thaliana* after *Pseudomonas syringae* infection

Andrea ROMERO-PÉREZ, Maarten AMEYE, Kris AUDENAERT, Els JM VAN DAMME

Content

Table S1. Primers used for CRISPR lines selection.

Table S2. Primers used for qPCR analysis.

Figure S1. Coding sequence of F-Box Nictaba gene (At2g02360).

Figure S2. Results of the selection of five F-Box Nictaba knock-out (KO) lines.

1. Supplementary Tables

Table S1. Primers used for CRISPR lines selection.

Target gene/Sequence	Forward primer (5'-3')	Reverse primer (5'-3')
Around mutation	P934 (CCACCGTTGATCCATAATGCA)	P874 (GCCGCATTACTTCAGAGGATT)
Around mutation (Sequencing)	P938 (ACAATGGAGCGTGAAGTAGAC)	-
Cas9	L205 (CTTGAGGATCTCCTTAACAA)	L206 (ATGTGCTTCTTGAAAGTCTC)

Table S2. Primers used for qPCR analysis.

Target gene	Forward primer (5'-3')	Reverse primer (5'-3')
F-box-Nictaba (FBN60) gene *(At2g02360)*	evd786 (TTGAGCTTGGGGAGTTCTTC)	evd787 (AGAGGATTTTAGCAGGTCGG)
PR1 gene *(At2g14610)*	evd1019 (GCTACGCAGAACAACTAAGAGG)	evd1020 (GCCTTCTCGCTAACCCACAT)
PR2 gene (At3g57260)	L374 (AAAACGGAGGCCGACAAG)	L375 (CGGAGGAGACGTATCAGTGGT)
WRKY70 gene *(At3g56400)*	evd811 (CATGGATTCCGAAGATCACA)	evd812 (CTGGCCACACCAATGACAA)
TIP41 *(At4g34270)* - Reference gene	evd729 (TGAACTGGCTGACAATGGAGTG)	evd730 (CATGAGCTTGGCATGACTCTCAC)
PP2A *(At1g13320)* - Reference gene	evd727 (TCCGAGATCACATGTTCCAAACTC)	evd728 (CCGTATCATGTTCTCCACAACCG)
UBC9 *(At4g27960)* - Reference gene	evd731 (TCCTACTTCATGTAGCGCAGGAC)	evd732 (TCCTCCAGAATAAGGGCTATCCG)

2. Supplementary Figures

>AT2G02360.1 CDS

ATGGGGAGAAAACGCAGAGTTA*AATCGGAGTCGTCTCCGTTCGATTCATTTCCGGAAGATTGCATCTC
CTACATAATCTCTTTTACAAATCCACGCGATGCGTGCGTCGCTGCTACGGTTTCGAAAACGTTTGAATCG
ACGGTGAAGTCAGATATTATATGGGAGAAGTTTCTTCCGGCGGATTATGAATCTCTGATTCCTCCATCG
CGAGTTTTCTCATCGAAGAAGGAGCTCTATTTCTCTCTCTGTAACGATCCTGTTCTATTCGACGATGACA
AAAAGAGCGTATGGTTAGAGAAAGCGAGTGGGAAGAGGTGTCTGATGTTATCTGCGATGAACCTCTCA
ATCATATGGGGAGATAATCCTCAGTATTGGCAATGGATTCCAATTCCTGAATCTAGGTTTGAAAAAGTA
GCGAAACTTCGCGATGTATGTTGGTTCGAGATTCGTGGCAGAACGAATACTCGTGTATTATCTCCAAGA
ACTCGTTACTCGGCTTATATTGTGTTCAAGGGAGTGGATAAATGTTATGGCTTTCAGAATGTGGCCATA
GAAGCTGCGGTAGGAGTGGTGGGACAGGAGCCTTCTAGAAGATTAATATGCTTTAGTGAAGCTATAAG
GAGGGGAAGGAGGAACGTTGTGAAACCTAAGCAGAGAGAAGATGGGTGGATGGAGATTGAGCTTGG
GGAGTTCTTCAATGATGGAGGAATAATGGATAATGATGAAATTGAGATGAGTGCTTTAGAGACTAAGC
AGCTCAATCGGAAGTGTGGCTTGATCATTCAAGGAATTGAAATCCGACCTGCTAAAATCCTCTGA

Figure S1. Coding sequence of F-Box Nictaba gene (At2g02360). The predicted cleavage site is marked with red. The 20 nucleotides forming the CRISPR spacer sequences are shown in green, and the 6 nucleotides composing the protospacer adjacent motifs (PAM) sequence in blue.

Knock-out line 1

KO1: 1 bp deletion

```
AT2G02360       ------------GAAGACGAAGACGAAGATACAAGCGTCGATCGATGGGGAGAAAACGCA 48
A1.P938         CTAGCTTCCGTGGAAGACGAAGACGAAGATACAAGCGTCGATCGATGGGGAGAAAACGCA 180
                            ************************************************

AT2G02360       GAGTTAAATCGGAGTCGTCTCCGTTCGATTCATTTCCGGAAGATTGCATCTCCTACATAA 108
A1.P938         GAGTTA-ATCGGAGTCGTCTCCGTTCGATTCATTTCCGGAAGATTGCATCTCCTACATAA 239
                ****** *****************************************************

AT2G02360       TCTCTTTTACAAATCCACGCGATGCGTGCGTCGCTGCTACGGTTTCGAAAACGTTTGAAT 168
A1.P938         TCTCTTTTACAAATCCACGCGATGCGTGCGTCGCTGCTACGGTTTCGAAAACGTTTGAAT 299
                ************************************************************
```

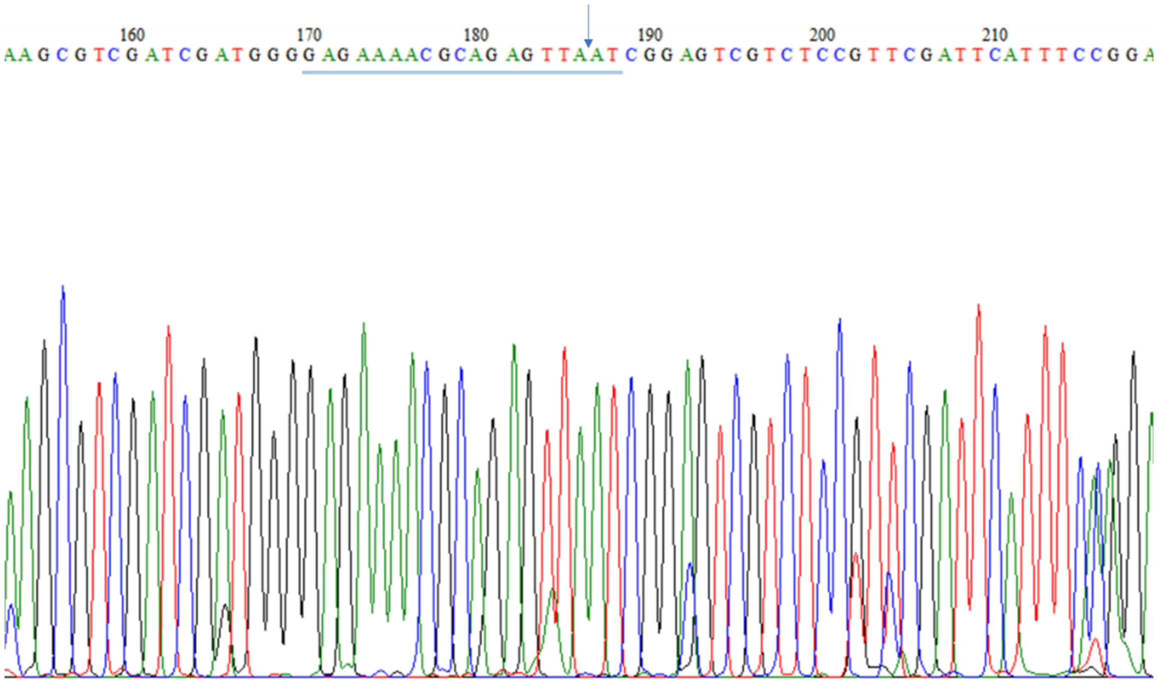

Knock-out line 2

KO2: 1 bp insertion

```
AT2G02360       -GAAGACGAAGACGAAGATACAAGCGTCGATCGATGGGGAGAAAACGCAGAGTTA-AATC 58
A2.P938         GGAAGACGAAGACGAAGATACAAGCGTCGATCGATGGGGAGAAAACGCAGAGTTAAAATC 180
                 ****************************************************** ****

AT2G02360       GGAGTCGTCTCCGTTCGATTCATTTCCGGAAGATTGCATCTCCTACATAATCTCTTTTAC 118
A2.P938         GGAGTCGTCTCCGTTCGATTCATTTCCGGAAGATTGCATCTCCTACATAATCTCTTTTAC 240
                ************************************************************

AT2G02360       AAATCCACGCGATGCGTGCGTCGCTGCTACGGTTTCGAAAACGTTTGAATCGACGGTGAA 178
A2.P938         AAATCCACGCGATGCGTGCGTCGCTGCTACGGTTTCGAAAACGTTTGAATCGACGGTGAA 300
                ************************************************************
```

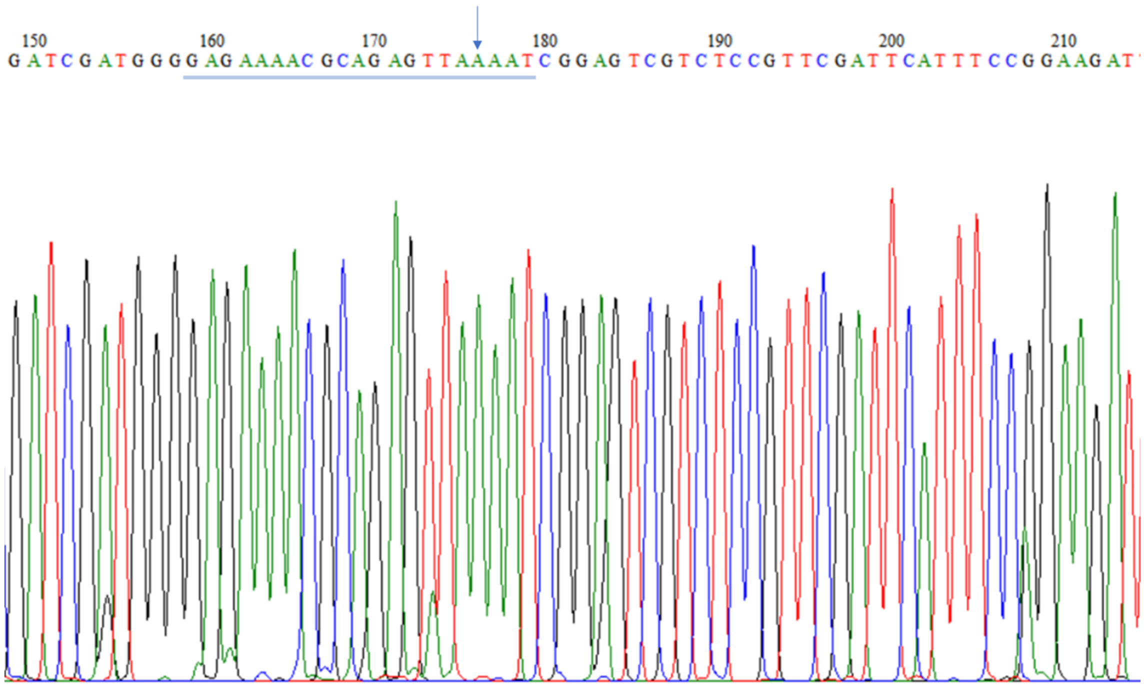

Knock-out line 3

KO3: 1 bp deletion

```
AT2G02360        -----------------GAAGACGAAGACGAAGATACAAGCGTCGATCGATGGGGAGAAA 43
A4.P938          TTACACTAGCTTCCGTGGAAGACGAAGACGAAGATACAAGCGTCGATCGATGGGGAGAAA 180
                                  *******************************************

AT2G02360        ACGCAGAGTTAAATCGGAGTCGTCTCCGTTCGATTCATTTCCGGAAGATTGCATCTCCTA 103
A4.P938          ACGCAGAGTTA-ATCGGAGTCGTCTCCGTTCGATTCATTTCCGGAAGATTGCATCTCCTA 239
                 *********** ************************************************

AT2G02360        CATAATCTCTTTTACAAATCCACGCGATGCGTGCGTCGCTGCTACGGTTTCGAAAACGTT 163
A4.P938          CATAATCTCTTTTACAAATCCACGCGATGCGTGCGTCGCTGCTACGGTTTCGAAAACGTT 299
                 ************************************************************
```

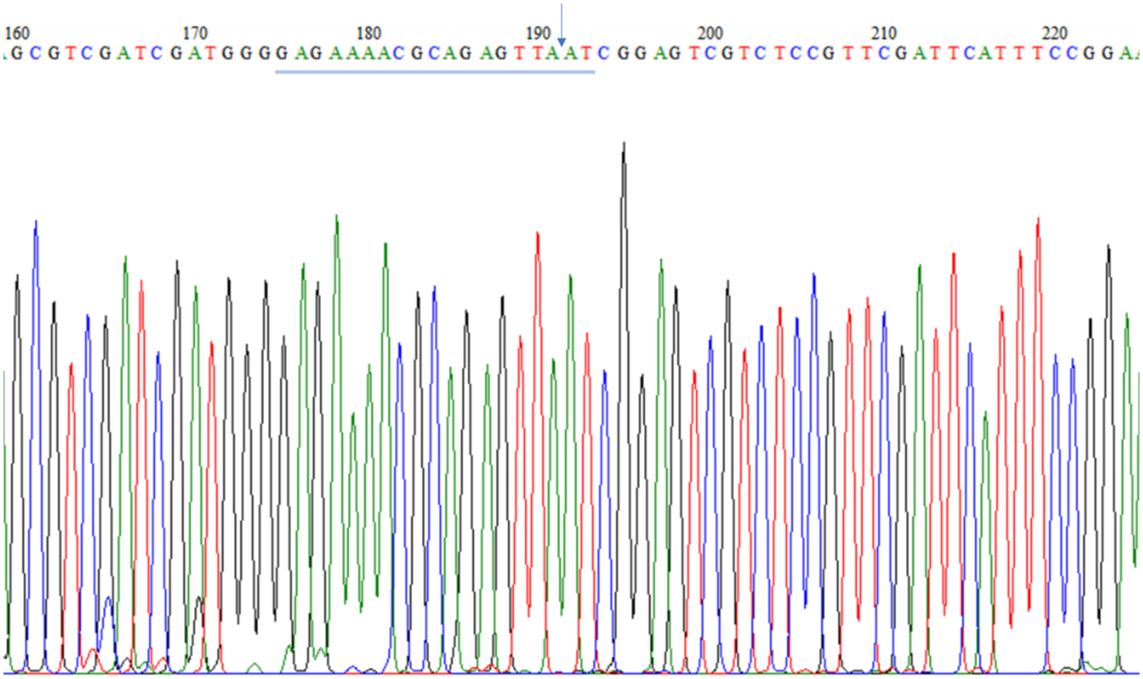

Knock-out line 4

KO4: 1 bp deletion

```
AT2G02360        -----------------GAAGACGAAGACGAAGATACAAGCGTCGATCGATGGGGAGAAA 43
A7.P938          TTACACTAGCTTCCGTGGAAGACGAAGACGAAGATACAAGCGTCGATCGATGGGGAGAAA 180
                                  *******************************************

AT2G02360        ACGCAGAGTTAAATCGGAGTCGTCTCCGTTCGATTCATTTCCGGAAGATTGCATCTCCTA 103
A7.P938          ACGCAGAGTTA-ATCGGAGTCGTCTCCGTTCGATTCATTTCCGGAAGATTGCATCTCCTA 239
                 *********** ************************************************

AT2G02360        CATAATCTCTTTTACAAATCCACGCGATGCGTGCGTCGCTGCTACGGTTTCGAAAACGTT 163
A7.P938          CATAATCTCTTTTACAAATCCACGCGATGCGTGCGTCGCTGCTACGGTTTCGAAAACGTT 299
                 ************************************************************
```

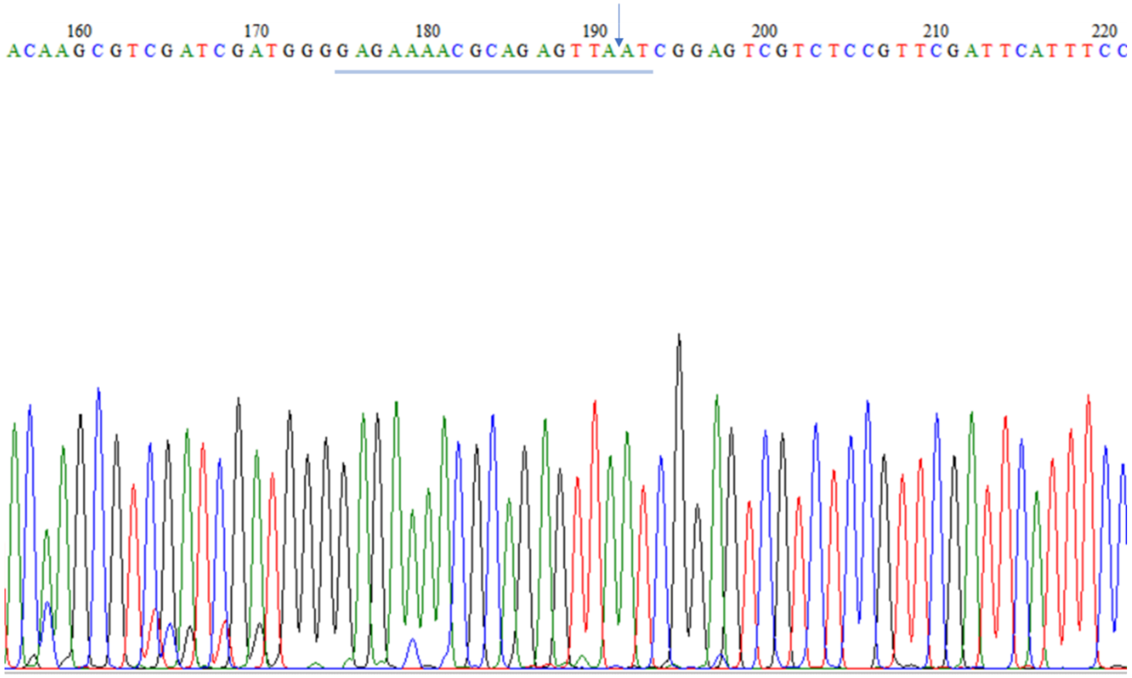

Knock-out line 5

KO5: 1 bp insertion

```
AT2G02360       ------------------GAAGACGAAGACGAAGATACAAGCGTCGATCGATGGGGAGAA 42
B10.P938        TTTACACTAGCTTCCGTGGAAGACGAAGACGAAGATACAAGCGTCGATCGATGGGGAGAA 180
                                  ******************************************

AT2G02360       AACGCAGAGTTA-AATCGGAGTCGTCTCCGTTCGATTCATTTCCGGAAGATTGCATCTCC 101
B10.P938        AACGCAGAGTTAAAATCGGAGTCGTCTCCGTTCGATTCATTTCCGGAAGATTGCATCTCC 240
                ************ ***********************************************

AT2G02360       TACATAATCTCTTTTACAAATCCACGCGATGCGTGCGTCGCTGCTACGGTTTCGAAAACG 161
B10.P938        TACATAATCTCTTTTACAAATCCACGCGATGCGTGCGTCGCTGCTACGGTTTCGAAAACG 300
                ************************************************************
```

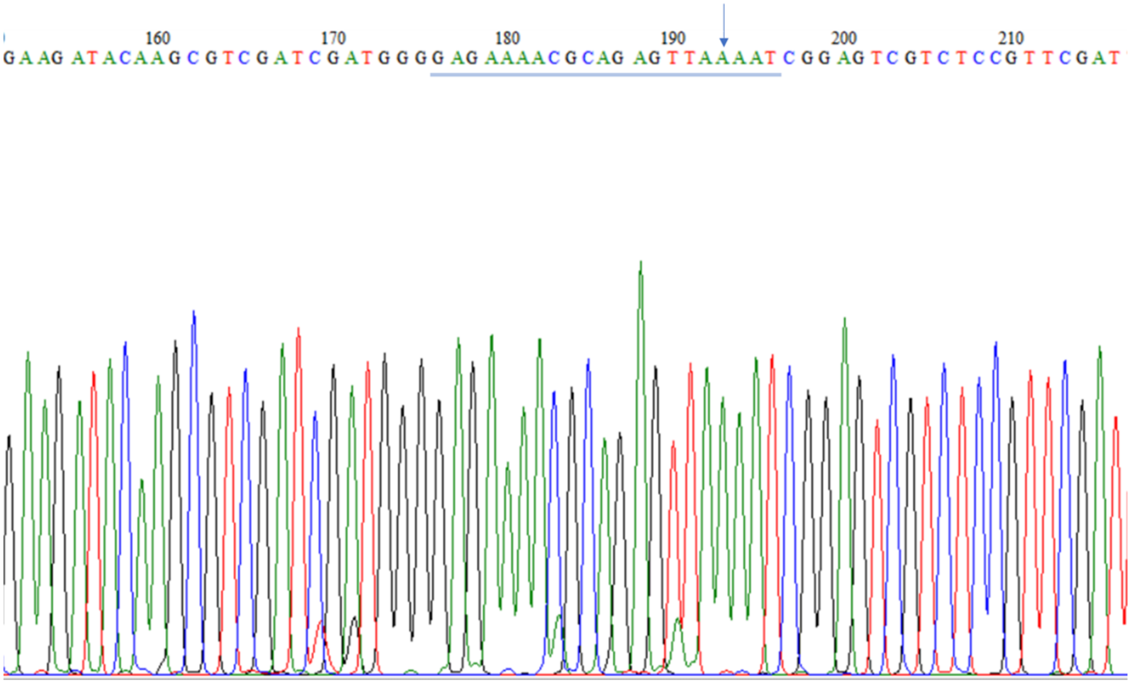

Figure S2. Results of the selection of five F-Box Nictaba knock-out (KO) lines. For every knock-out line: sequence alignment of the WT F-Box Nictaba sequence with the knock-out F-Box Nictaba sequence (top); sequencing chromatograms indicating the CRISPR spacer sequences (blue) and the predicted cleavage sites (arrow).
